# Clinical application study of a novel fully automatic erythrocyte osmotic fragility analysis system

**DOI:** 10.1016/j.bbrep.2025.102224

**Published:** 2025-09-01

**Authors:** Zixin Deng, Yi Li, Zhizhi Xiang, Yi Liu, Jingbo Tang, Song Zhang, Guangchao Zang, Yingying Gao, Lei Ma

**Affiliations:** a Department of Medical Laboratory, Affiliated Hospital of Chongqing Medical and Pharmaceutical College, Chongqing Medical and Pharmaceutical College, Chongqing, China; b Department of Medical Laboratory, Banan Hospital Affiliated to Chongqing Medical University, Chongqing, China; c Biomedical Innovation and Entrepreneurship Practice Base, Lab Teaching & Management Center, Chongqing Medical University, Chongqing, China; d Chongqing Lifotronic Innovation Biotechnology Co.,Ltd., Chongqing, China; e School of Medical Technology, Chongqing Medical and Pharmaceutical College, Chongqing, China

**Keywords:** Erythrocyte fragility test (EFT), Thalassemia screening, Automated analyzer, Accuracy, Instrument performance

## Abstract

**Background:**

Existing erythrocyte osmotic fragility test (EFT) methods are constrained by subjectivity, poor reproducibility, and lack of standardization, limiting their clinical utility in thalassemia screening. This study evaluates the RA-800 Plus, a novel fully automated EFT analyzer, as a scalable tool for thalassemia screening and aims to establish reference intervals for healthy individuals. By addressing the key limitations of manual EFTs, this work seeks to promote methodological innovation and enable standardized, high-throughput screening in diverse clinical settings.

**Methods:**

EFTs were performed on 273 healthy adults via an RA-800 Plus analyzer. The light scattering turbidity method was employed to establish a reference interval within the normal range. The neonatal samples were tested in the same way to determine the newborn-specific range. Moreover, 97 samples underwent dual tests of RA-800 Plus analysis and the gold standard (genetic testing) to evaluate the diagnostic performance. A total of 103 pairs of samples were detected and compared via an automated system and the traditional manual direct colorimetric method, further verifying the consistency of the two methods. The effects of anticoagulant type, sample storage, and rewarming were also investigated.

**Results:**

RA-800 Plus demonstrated high reliability, with minimal influence from anticoagulants and sample handling conditions. The analyzer showed an 84 % detection rate for β-thalassemia, indicating superior effectiveness for β-thalassemia screening. The test results were consistent with the manual method (Kappa ≥0.6). ROC curve analysis confirmed the suitability of both methods for thalassemia screening, with AUCs of 0.91 for β-thalassemia and 0.72 for α-thalassemia.

**Conclusions:**

The RA-800 Plus offers a fully automated, reliable, and clinically viable alternative for thalassemia screening, particularly for β-thalassemia. Its stability, accuracy, and ease of use make it a valuable tool for improving thalassemia diagnostics.

## Highlights

• **High Accuracy for β-Thalassemia Screening**: RA-800 Plus automated analyzer achieved AUC 0.96 and 86% detection rate for β-thalassemia screening.
• **Stability Across Anticoagulants and Handling Conditions**: Results varied ≤2% across heparin, EDTA-K₂, and sodium citrate, stable under storage and rewarming.
• **Clinically** **Validated Reference Intervals**: Established erythrocyte fragility ranges (65–86% adults, 16.28–67.25% neonates) consistent with manual tests (Kappa ≥0.4).

## 1 Introduction

Thalassemia is a widely distributed single-gene hereditary disorder caused by defective synthesis of α- or β-globin chains, leading to an imbalance between globin subunits, ineffective erythropoiesis, hemolysis, and iron overload–related complications. Owing to its phenotypic heterogeneity, early and accurate screening is critical for clinical management and reducing the population burden [[1], [2], [3]], mitigating the risk of complications caused by iron overload, and lowering the incidence of thalassemia [4,5]. Conventional screening tools such as mean corpuscular volume (MCV), mean corpuscular hemoglobin (MCH), and electrophoresis are commonly used but often lack specificity and standardization, particularly in primary care settings. Erythrocyte osmotic fragility (EFT), which reflects the biomechanical stability of red blood cells (RBC), offers a physiologically relevant and cost-effective alternative for thalassemia screening [6,7].

Currently, the most commonly used methods for testing the EFT include osmotic fragility tests (OFTs), mechanical fragility tests [8,9], flow cytometry [[10], [11], [12], [13], [14], [15]], and microscopic observation [16,17]. Each method has distinct advantages and limitations [18,19]: mechanical and flow-based assays offer high sensitivity and mechanistic depth but require expensive instrumentation and technical expertise; microscopic and classical manual OFTs are more accessible but suffer from low throughput and subjectivity.

In clinical practice, particularly in primary care and resource-constrained environments, manual OFTs continue to be the most widely adopted due to their simplicity. However, this approach is associated with significant operator dependency, limited processing capacity, and considerable variability across laboratories. In addition, environmental conditions and subjective endpoint identification further compromise consistency, making manual EFTs difficult to standardize or scale for population screening, and limiting their alignment with genetic diagnosis. Automated turbidimetric EFT analyzers have emerged to overcome these limitations, offering quantitative output, improved repeatability, and simplified workflows. Despite the recognized value of OFTs, no fully automated system has undergone rigorous clinical validation across real-world samples, with robust benchmarking against genetic and manual gold standards. The lack of population-specific reference intervals, unclear preanalytical stability, and poor reproducibility have prevented wider clinical deployment [20].

To bridge this gap, we evaluated the RA-800 Plus, a fully automated EFT analyzer based on nephelometric turbidity measurements. Our objectives were threefold: to establish age-specific reference intervals, to assess analytical robustness under common clinical conditions, and to benchmark its diagnostic performance against both manual testing and thalassemia genotyping. This work represents the first comprehensive clinical validation of an EFT analyzer, incorporating [1] the development of population-specific reference ranges [2], head-to-head comparisons with genetic and manual standards, and [3] a systematic evaluation of preanalytical variables including anticoagulant types and sample storage. Collectively, our findings support the advancement of EFT into a reliable, automated, and scalable tool for standardized thalassemia screening.

## 2 Materials and methods

### 2.1 Instruments and working principle

The EFT in this study was performed using the RA-800 Plus automatic analyzer (Shenzhen Shenzhen Pumen Technology Co., China). The instrument determines RBC fragility by nephelometric turbidity measurements, which detect changes in scattered light intensity due to cell lysis in hypotonic solution.

Normal biconcave RBCs placed in hypotonic saline swell due to osmotic pressure and eventually rupture, resulting in hemolysis. As the number of intact cells decreases, the turbidity of the suspension diminishes correspondingly. The RA-800 Plus employs a 650 nm red laser to measure the scattered light intensity before (A_0_) and after (A_1_) a 50-s reaction period. The hemolysis rate (%) is calculated as follows:

$$Hemolysisrate\left(\%\right)=\frac{\left(A0-A1\right)}{A0}\times 100\%$$

**Fig. 1:**
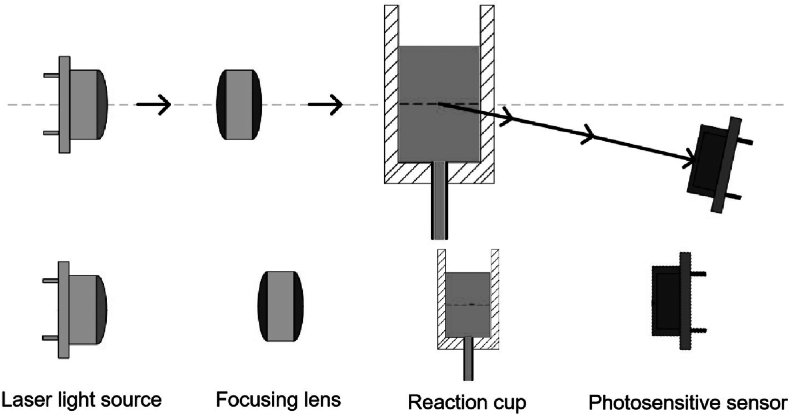
Optical system of the RA-800 plus.

The light signal is captured by a photosensitive sensor, and the calculation is based on relative light intensity changes. A schematic representation of the optical system is provided in Fig. 1.

The nephelometric turbidity method was chosen due to its superior objectivity, quantifiability, and minimal dependence on subjective interpretation—offering distinct advantages over visual or manual EFT methods.

### 2.2 Patients

From November 2023 to June 2025, whole blood samples were collected at Chongqing Chenjiaqiao Hospital and analyzed via the fully automated erythrocyte osmotic fragility analyzer (RA-800 Plus, Shenzhen Pumen Technology Co., Ltd.). A total of 273 healthy adult subjects (covering all age groups and both sexes, excluding newborns) were included to establish the reference interval for erythrocyte fragility. In addition, 52 neonatal samples were independently collected to define the reference range for newborns [21]. To assess the clinical performance of the RA-800 Plus, 97 samples underwent parallel testing with genetic analysis as the gold standard [22]. Furthermore, 103 samples were tested via both the RA-800 Plus and the manual direct colorimetry method to evaluate their methodological agreement. All experimental methods were reviewed by the Ethics Committee, and the submitted research protocol and other materials of the project complied with the principles of medical ethics and the requirements of the Helsinki Declaration. The research design conforms to relevant laws and regulations. The research protocol design of this project has been approved, and the approval number is CJQYY-ECKY-2022006.

### 2.3 Direct colorimetry

The direct colorimetric method, also known as the manual method, involves preparing 14 tubes containing different concentrations of NaCl (ranging from 2.0 to 7.2 g/L with an interval of 0.4 g/L). Using a dry sterilized syringe, 1 mL of venous blood was collected from the subject and 1 drop of whole blood was added to each tube, followed by gentle mixing. The same procedure was applied to collect blood from healthy controls, which was placed in control tubes. All the tubes were then left at room temperature for 2 h. Hemolysis was observed starting from the highest concentration tube. At the onset of hemolysis: the supernatant begins to appear slightly pink, while a significant number of undissolved RBC remain at the bottom of the tube. In completely hemolyzed tubes: the entire solution turned dark red, and no RBC or residual debris was visible at the bottom. The corresponding sodium chloride concentration was recorded. Based on the reference ranges (onset of hemolysis: 3.8–4.6 g/L; complete hemolysis: 2.8–3.2 g/L), the presence of increased or decreased osmotic fragility was determined.

### 2.4 Sample preprocessing study

Anticoagulant: The three types of anticoagulant blood collection tubes used in this study were EDTA-K2, heparin, and sodium citrate tubes, which are commonly employed in clinical practice. Experimental samples were obtained from fresh whole blood from healthy donors, ensuring that all blood in each experimental group came from the same donor to avoid individual differences affecting the results. The collected anticoagulated whole blood was aliquoted into anticoagulant tubes pretreated with heparin, EDTA-K2, or sodium citrate, respectively. To ensure the reproducibility of the experiment, each blood sample corresponding to each anticoagulant was collected at least twice (n ≥ 2) for subsequent testing. Both manual and nephelometric turbidity test results for each anticoagulant group were recorded, and two repeated tests were conducted to minimize random error. The differences between the manual and turbidimetric methods were analyzed and compared.

Rewarming process and storage stability: To evaluate the impact of the refrigeration and rewarming processes on the EFT results, two fresh whole-blood samples were first collected via the conventional venipuncture method. After collection, each sample was immediately mixed to ensure uniformity, followed by five repeated EFT via the turbidimetric method. The average value was recorded. After the two samples were placed in an environment controlled at 2–8 °C for 1 h, they were removed, rewarmed to room temperature, and mixed thoroughly. Five repeated EFTs were immediately conducted again, and the average value of post-rewarming was recorded. The absolute deviation between the average erythrocyte fragility values before and after refrigeration was calculated. The samples whose deviation exceeded 2 % were left at room temperature to recover. EFTs were conducted every 15 min after gently mixing the samples until the deviation from the pre-refrigeration results decreased to no more than 2 %.

Additionally, to assess the impact of different anticoagulants (EDTA-K2, heparin, and sodium citrate) under various storage conditions on the EFT results, three fresh whole blood samples were collected and placed them in the respective anticoagulant tubes. Each sample was tested three times via the nephelometric turbidity method, and the initial average value was recorded. The blood samples in each anticoagulant tube were subsequently divided into two groups: one group was stored at 2–8 °C, while the other was kept at room temperature. Every 2 h, two EFTs were conducted on the refrigerated and room-temperature samples, and the average values were calculated. The refrigerated samples were rewarmed to room temperature before testing to ensure comparability of the results. It is worth noting that sodium citrate was not included in the stability assessment, as its calcium-chelating properties significantly alter the plasma ionic strength and red cell osmotic response, which may interfere with the nephelometric measurement principle of the RA-800 Plus analyzer [23].

### 2.5 Establishment of reference intervals

Study Subjects, Inclusion and Exclusion Criteria, and Analysis Indicators: The inclusion criteria for healthy reference individuals were established according to the NCCLS C28-A2 guidelines. All the samples were obtained from individuals who underwent routine physical examinations and had no physiological indicators that could affect the test results, no history of medication use or dietary therapy, and no organ diseases involving the cardiovascular, pulmonary, renal, hepatic, or pancreatic systems. Additionally, individuals with recent blood transfusions or surgeries were excluded, and women who were not menstruating, pregnant, or lactating were excluded. Samples with hemolysis, jaundice, or inadequate storage conditions were also excluded. The analyzer's direct readout of EFT results under different conditions was used as the analysis indicator [24].

### 2.6 Statistical analysis methods

A total of 273 fresh whole blood samples from healthy adults (115 males and 158 females, aged 1–90 years) and 52 samples from healthy neonates (aged <28 days) were collected to establish age-specific reference intervals via the nephelometric turbidity method. The outliers were identified via the D/R ratio, where D represents the difference between the maximum (or minimum) value and the second-largest (or second-smallest) value, and R represents the range of the data set. When D ≥ 1/3R, the extreme value was considered an outlier and excluded. In cases where outliers clustered asymmetrically, further evaluation was performed to determine whether exclusion was appropriate.

Group comparisons were conducted via the z-test. If no statistically significant difference was found, a combined reference interval was reported; otherwise, separate reference intervals were established for each group.

All datasets were complete, with no missing values identified. Consequently, no imputation techniques were required.

### 2.7 Comparison and validation of clinical performance indicators

This study adopted a retrospective cohort design based on real-world clinical workflows, where all samples had undergone genetic testing prior to RA-800 Plus analysis. A strict double-blind protocol was maintained between operators of the two methods. This approach ensures practical relevance while providing robust evidence for the clinical applicability of the analyzer.

### 2.8 Gene testing

To establish a case sample set, 97 whole blood samples were collected from clinical centers in Sichuan, Chongqing, and Guizhou. All samples first underwent thalassemia genetic testing, followed by erythrocyte fragility assessment via the RA-800 Plus analyzer. The testing process was conducted under a strict double-blind protocol, ensuring that genetic testing personnel and EFT operators were mutually blinded. The sample set included 33 cases of α-thalassemia, 44 cases of β-thalassemia, no cases of αβ-compound thalassemia (due to its rarity in the study population), and 20 genetically negative controls. This retrospective cohort design, grounded in real-world clinical workflows and rigorous blinding, supports the robustness and clinical relevance of the analyzer's performance evaluation. Peripheral blood DNA was extracted to detect various gene mutations and deletion types associated with thalassemia, particularly α-thalassemia gene deletions (--SEA type deletion, α-3.7 and α-4.2 gene deletions, and non-deletion mutations such as αCS and αWS) and β-thalassemia gene mutations (CD41-42(-TTCT) mutation, CD17(A > T) mutation, IVS-II-654(C > T) mutation, CD43(G > T) mutation, along with other common point mutations or insertion mutations). The experimental process strictly followed the standard procedures and reagents specified by the instrument.

### 2.9 Clinical statistical validation

To validate the performance of the RA-800 Plus erythrocyte fragility analyzer in different thalassemia samples and its concordance with gene testing, this study conducted nephelometric turbidity tests on samples identified as α-thalassemia, β-thalassemia, and α+β-thalassemia, as well as negative samples. Both normal distribution and non-parametric percentile methods were used. Normal distribution analysis was employed to determine specific reference intervals, calculate the sample size, minimum and maximum values, means, medians, standard deviations, skewness, and kurtosis. During this process, positive and negative samples were classified via a 95 % confidence interval, establishing reference interval ranges for each type of thalassemia and identifying specific upper limit values (based on right-sided analysis of the 95 % confidence interval). The non-parametric percentile method (CLSI C28-A3) was used to estimate reference upper limit values for different thalassemia types. Specific values were estimated through a 90 % confidence interval to further optimize the accuracy of the test results. This approach effectively handles non-normally distributed data and provides more robust statistical results [25].

### 2.10 Comparison of direct colorimetry results

A total of 103 valid whole blood samples with EDTA-K2 anticoagulant were collected. The erythrocyte fragility of the samples was tested via both the RA-800 Plus erythrocyte fragility analyzer and the manual method. The nephelometric turbidity method for detecting erythrocyte fragility utilizes preset testing parameters, with determination based on reference values (65 %–86 %). The manual EFTs were conducted according to standard laboratory procedures, and the results were qualitatively assessed using the same reference range and standards.

The direct colorimetry utilized EDTA-K2 anticoagulant, with reference intervals established in accordance with Clinical Laboratory Operating Procedures (initial hemolysis: 4.2–5.2 g/L; complete hemolysis: 3.2–3.6 g/L). Each sample was analyzed qualitatively on the basis of the reference standards of both the RA-800 Plus and manual methods to assess whether it fell within the normal fragility range. The concordance between the two testing methods was subsequently evaluated.

## 3 Results

### 3.1 Anticoagulant study

To evaluate the impact of different anticoagulants on erythrocyte fragility detection values, three commonly used anticoagulants were selected: heparin, EDTA-K2, and sodium citrate. Whole blood from the same donor was aliquoted into tubes pretreated with each anticoagulant. To ensure reproducibility, each blood sample for each anticoagulant was collected at least twice (n ≥ 2) for subsequent testing. Both manual and nephelometric turbidity test results were recorded, with two replicate tests conducted to minimize random error.

**Fig. 2:**
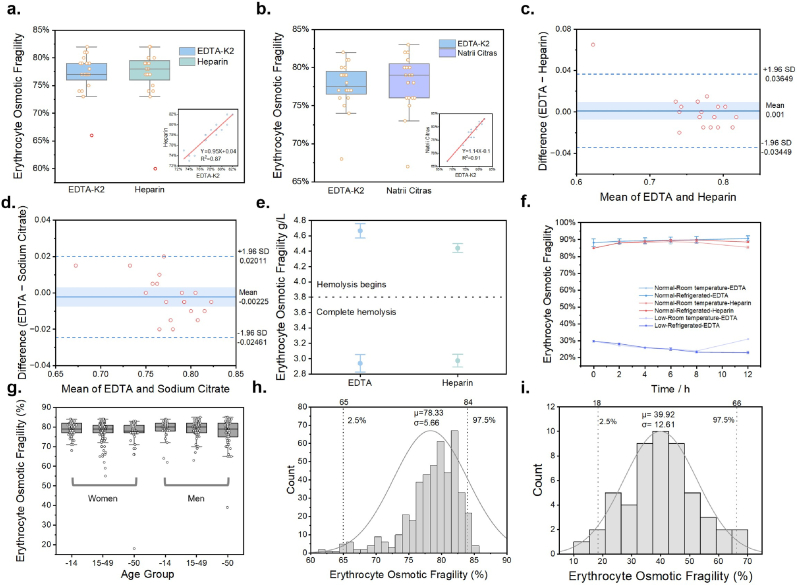
(a–b) Comparison of nephelometric fragility test results for normal samples with different anticoagulants: (a) EDTA vs. Heparin (b) EDTA vs. sodium citrate. (c) Bland–Altman plot showing agreement between EDTA and heparin anticoagulants in EFT measurements (d) Bland–Altman plot showing agreement between EDTA and sodium citrate anticoagulants in EFT measurements. (e) Manual method results for different anticoagulants. (f) Sample storage stability testing. (g) Analysis of erythrocyte fragility results in different age groups: men (right) and women (left). (h) Normal distribution of EFTs results in the healthy population (excluding neonates). (i) Normal distribution of EFTs results in healthy neonates.

As shown in Fig. 2a-d, there is a minimal difference in the EFTs results of the normal samples among the different anticoagulants. The average error in fragility results between the heparin and sodium citrate groups, compared with the EDTA-K2 group, was 2 %. After excluding outliers, the scatter plot of the fragility results under different anticoagulant conditions revealed that the data points were mostly evenly distributed around a line with a slope of 1. The linear regression coefficients for the heparin/EDTA-K2 and sodium citrate/EDTA-K2 groups were 0.87 (Figs. 2a) and 0.91 (Fig. 2b), respectively, with slopes of 0.95 and 1.14. These findings suggest that the nephelometric method produces minimal differences in fragility test values across different anticoagulants.

The manual method, as shown in Fig. 2e, revealed a slight difference in apparent fragility values between the anticoagulants. Specifically, the fragility of the heparin group was found to be 0.4 units lower than that of the EDTA-K2 group.

### 3.2 Sample preprocessing stability

To evaluate the impact of refrigeration and rewarming on erythrocyte fragility, two fresh whole blood samples were collected via venipuncture and tested both before and after refrigeration. The samples were cooled to 2–8 °C for 1 h and then rewarmed to room temperature. Each sample was tested five times under both pre-refrigeration and post-rewarming conditions. The average fragility values were recorded, and the absolute deviation between these values was calculated to assess any changes induced by these processes.

The results indicate that the variations in mixing cycles, warming times and frequencies, and different storage conditions led to fluctuations in the RA-800 Plus fragility test results of up to 2 %. However, these fluctuations were observed to be random, with no consistent pattern or systematic bias. As shown in Fig. 2f, the nephelometric turbidity test results remained stable across various storage conditions and durations, suggesting that the sample preprocessing process, including refrigeration and rewarming, did not significantly impact the RA-800 Plus test outcomes.

### 3.3 Reference intervals

The reference interval for erythrocyte fragility was established on the basis of the test results of 253 healthy individuals (115 males and 138 females, aged 1–90 years) from the Chongqing region, using EDTA-K2 as an anticoagulant (Fig. 2g). The fragility test results followed a normal distribution, with a mean of 78.33 % and a standard deviation (σ) of 5.66 %. Using the 2.5th to 97.5th percentile range, the reference interval was determined to be 65 %–84 %. No significant differences were observed across age groups (excluding preterm newborns) or genders (P > 0.05) (Fig. 2h).

Based on the above results regarding anticoagulant effects, the reference range for heparin- and sodium citrate-anticoagulated samples was estimated as 67 %–86 %. Taking into account anticoagulant-induced variations, the recommended reference range for the Pumen Technology Co. measurement kit (turbidimetric method) is 65 %–86 %. To account for differences in RBC composition between neonates and adults, a separate reference interval was established for neonates. Fragility tests on 23 normal neonatal samples yielded a reference range of 18 %–66 % (Fig. 2i).

### 3.4 Clinical performance validation

To evaluate the clinical performance of the nephelometric method, we retrospectively analyzed 97 genetically confirmed thalassemia cases from three regions (Sichuan, Chongqing, and Guizhou), ensuring demographic diversity and real-world representativeness. To address the issue of class imbalance, the Synthetic Minority Over-sampling Technique (SMOTE) was applied prior to ROC curve reconstruction.

**Fig. 3:**
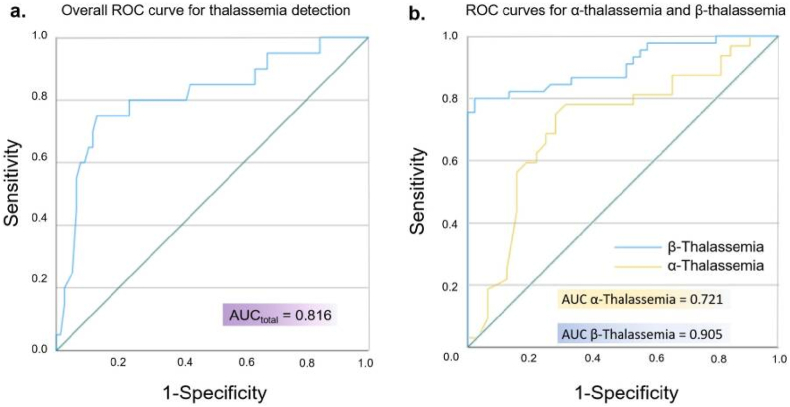
ROC curves for evaluating direct colorimetry (a) and diagnostic (b) tests.

As shown in Fig. 3a, the nephelometric test results were compared against the genetic gold standard across all subjects (combining α- and β-thalassemia with controls). After SMOTE-based augmentation of the binary classification, the method achieved an AUC of 0.816. Fig. 3b further focused on the thalassemia-positive group, where SMOTE was used again to balance α- and β-subtypes. The nephelometric method yielded an AUC of 0.905 for β-thalassemia and 0.721 for α-thalassemia, indicating better discriminatory power for the β-subtype.

In addition to ROC analysis, we evaluated inter-method agreement using contingency tables. As shown in Table 1, the nephelometric method demonstrated substantial concordance with the direct colorimetry method (κ = 0.651). Relative to genetic testing, the RA-800 Plus achieved a sensitivity of 84.1 %, specificity of 80.0 %, and overall accuracy of 82.8 % for β-thalassemia (Table 2). For α-thalassemia, while the sensitivity was lower (42.4 %), the method maintained acceptable specificity (80.0 %) and a total accuracy of 56.6 % (Table 3), supporting its potential for preliminary screening.

**Table 1:** Two methods of diagnosis consistency kappa test results.

	Direct colorimetry positive	Direct colorimetry negative	Kappa	Result
RA-800 plus positive	42	10	0.651	Substantial Agreement
RA-800 plus negative	8	43

**Table 2:** β-Thalassemia.

	Positive genetic diagnosis	Negative genetic diagnosis	Sensitivity (%)	Specificity (%)	Accuracy (%)
RA-800 plus positive	37	4	84.1	80	82.8
RA-800 plus negative	7	16

**Table 3:** α-Thalassemia.

	Positive genetic diagnosis	Negative genetic diagnosis	Sensitivity (%)	Specificity (%)	Accuracy (%)
RA-800 plus positive	14	4	42.4	80	56.6
RA-800 plus negative	19	16			

### 3.5 Rationale for selecting the RA-800 plus analyzer

1) Automation and Standardization: Traditional OFTs are highly dependent on manual operation and visual interpretation, leading to poor reproducibility and limited scalability. RA-800 Plus provides a fully automated solution with predefined reaction times and detection thresholds, eliminating operator subjectivity and allowing for high-throughput screening. This standardization is critical for consistent clinical application, particularly in large-scale screening settings.
2) Analytical Superiority: The RA-800 Plus utilizes nephelometric detection rather than the more commonly used transmission-based colorimetry. This method offers superior specificity by minimizing interference from sample turbidity, hemolysis artifacts, and cellular debris. Additionally, our results confirmed its robustness against variations in anticoagulant type and sample handling—factors that often undermine OFT reliability.
3) Clinical Feasibility: Unlike flow cytometry or mechanical fragility tests, which are costly and technically demanding, the RA-800 Plus is compact, cost-efficient, and user-friendly. Its 1-min detection cycle, minimal sample preparation requirements, and reagent simplicity make it suitable for implementation in routine clinical workflows, especially in primary care and resource-limited settings.

## 4 Discussion

This study demonstrates that the RA-800 Plus nephelometric method offers significant advantages over the manual direct colorimetry method, particularly in its robustness to ionic strength variations introduced by different anticoagulants. While the manual method showed minor shifts in fragility values across anticoagulants, these differences were likely caused by ionic strength–induced changes in hemolysis thresholds. In contrast, the nephelometric method maintained high consistency, with less than 2 % variation across all groups. Sample preprocessing, including refrigeration and rewarming, did not significantly affect the test outcomes. The fluctuations remained within acceptable limits, suggesting that the RA-800 Plus provides stable results even under routine laboratory variations. This robustness reinforces its suitability for use in diverse clinical settings.

Furthermore, age-specific reference intervals were established using 273 adults and 52 neonates. Among adults, no significant differences were observed by age or sex, whereas neonates exhibited notably lower fragility values, likely attributable to higher membrane elasticity and the predominance of fetal hemoglobin, suggesting the need for subtype-specific thresholds in neonatal screening. Validation against genetic testing revealed that the RA-800 Plus demonstrated strong diagnostic performance for β-thalassemia (AUC = 0.905) and acceptable accuracy for α-thalassemia (AUC = 0.721). High specificity across both subtypes minimizes false positives, making it a valuable tool for large-scale screening.

## 5 Conclusion

The RA-800 Plus fully automated erythrocyte fragility analyzer represents a notable advancement in EFTs, offering superior accuracy, stability, and reduced susceptibility to anticoagulant-induced ionic strength variations. We demonstrated that the RA-800 Plus is maintains consistent performance across different anticoagulants, with only ∼2 % variation between heparin and sodium citrate compared to EDTA-K2, underscoring the robustness of its nephelometric detection principle. A clinically relevant reference interval for erythrocyte fragility (65–86 %) was established through the analysis of a large cohort of clinical samples, which closely aligned with genetic testing results, particularly for β-thalassemia. Furthermore, the system showed strong resistance to common preanalytical variables, ensuring reliable operation in diverse clinical settings. This analyzer demonstrated superior efficacy in detecting β-thalassemia, yet it exhibited potentially lower sensitivity for α-thalassemia detection. To address this, future developments will focus on integrating additional hematological parameters—such as erythrocyte indices—and exploring combined screening strategies to increase diagnostic accuracy and broaden clinical applicability. Overall, the RA-800 Plus introduces a more standardized, efficient, and reproducible approach to EFT, with clear potential to streamline clinical workflows and set a new benchmark for next-generation hematological diagnostics.

## CRediT authorship contribution statement

**Zixin Deng:** Writing – review & editing, Writing – original draft, Investigation, Formal analysis, Data curation. **Yi Li:** Writing – review & editing, Writing – original draft, Formal analysis, Data curation. **Zhizhi Xiang:** Writing – original draft, Methodology, Investigation, Data curation. **Yi Liu:** Software, Investigation. **Jingbo Tang:** Software, Resources, Methodology. **Song Zhang:** Software, Resources. **Guangchao Zang:** Writing – review & editing, Supervision, Conceptualization. **Yingying Gao:** Writing – review & editing, Supervision, Funding acquisition. **Lei Ma:** Supervision, Funding acquisition.

## Declaration of competing interest

The authors declare that they have no known competing financial interests or relevant relationships that could have appeared to influence the work reported in this paper.

## Data Availability

Data will be made available on request.
